# Supplementary material for: Experimental warming causes mismatches in alpine plant-microbe-fauna phenology
Source: Nat Commun. 2023 Apr 15;14:2159. doi: 10.1038/s41467-023-37938-3 (PMC10105701; doi:10.1038/s41467-023-37938-3)
Supplement: Supplementary file 1 — Supplementary Information File [file 41467_2023_37938_MOESM1_ESM.pdf]

Supplementary information

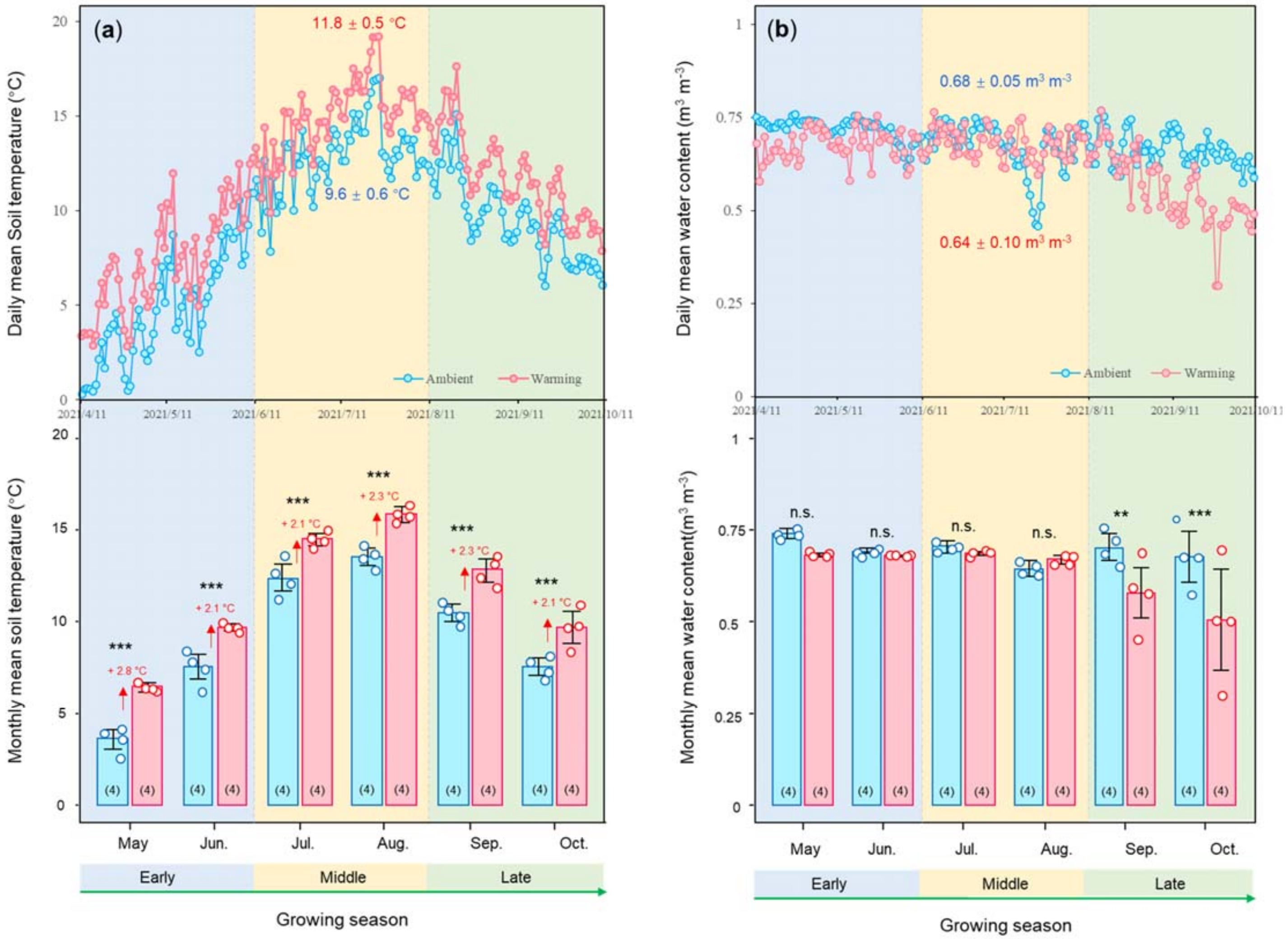

Supplementary Fig. 1 Dynamics of soil temperature and soil water content at 5 cm soil depth during the growing season of 2021. (a) Daily mean

soil temperature dynamics (top panel), and effects of warming on monthly mean soil temperature (n =4) in ambient and warmed plots (bottom panel). The mean soil temperature of the growing season at ambient and warming treatments was 9.6 ± 0.6°C and 11.8 ± 0.5°C, respectively. The warmed plots achieved a warming effect of about 2.2°C during the growing season, and the warming effect was consistently significant (ranging from 2.1°C to 2.8°C) across months. **(b)** Daily mean soil water content (volumetric) dynamics (top panel), and effects of warming on monthly mean water content (n =4) in ambient and warmed plots (bottom panel). The mean soil water content during the growing season in the ambient and warmed plots was 0.68 ± 0.05 m^3 m^{-3} and 0.64 ± 0.10 m^3 m^{-3}, respectively. In warmed plots, soil mean water content was slightly decreased (by 0.06 m^3 m^{-3}) across the growing season. In bottom panels **(a, b)**, data shown are mean values ± SD, and jittered points represent individual plots for each climate treatment (blue for ambient plots, n = 4; red for warmed plots, n = 4). The date were analyzed by linear mixed effect models, and multiple comparisons were adjusted by FDR corrections to test the effects of warming on soil temperature and soil water content, separately for each month. Significant differences between ambient and warming climates are indicated by ** = $P < 0.01$, *** = $P < 0.001$.

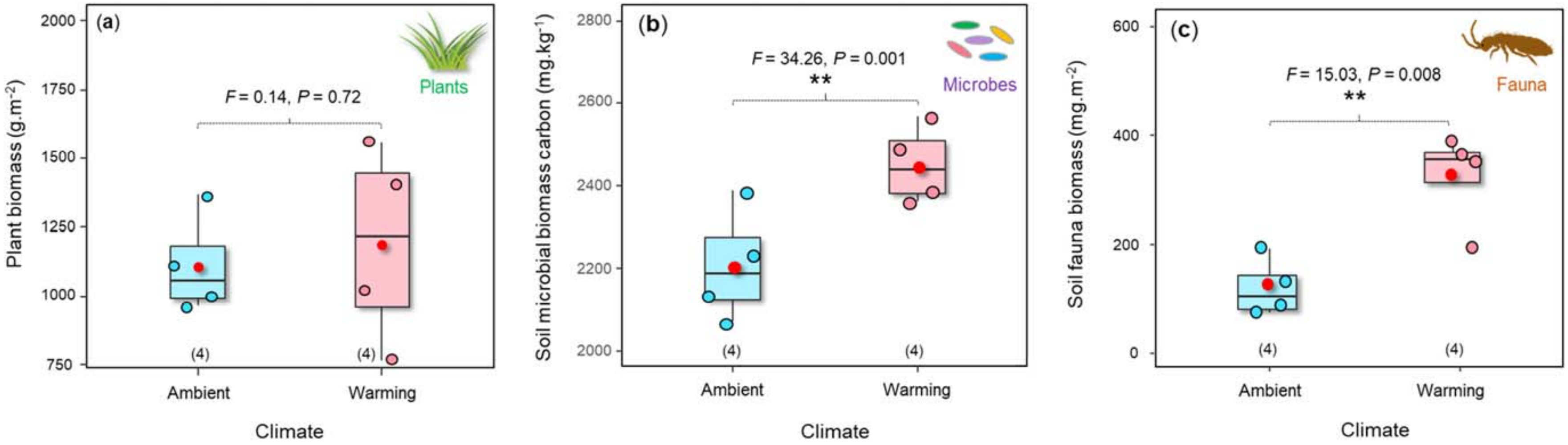

Supplementary Fig. 2 Responses of the potential explanatory variables. Effects of climate (ambient vs. experimental warming) on **(a)** plant biomass (n = 4), **(b)** soil microbial biomass carbon (n = 4), and **(c)** soil fauna biomass (n = 4). Data were analysed using linear mixed effects models (see Methods for details). Significant differences between ambient and warming climates are based on two-sided tests for multiple comparisons by FDR corrections, and indicated by ** = $P < 0.01$). Box centre lines represent the median, box limits represent the upper and lower quartiles, whiskers represent the 1.5 × interquartile range from the 25th and 75th percentiles, red dots represent the mean, and jittered points represent biologically independent samples for each group.

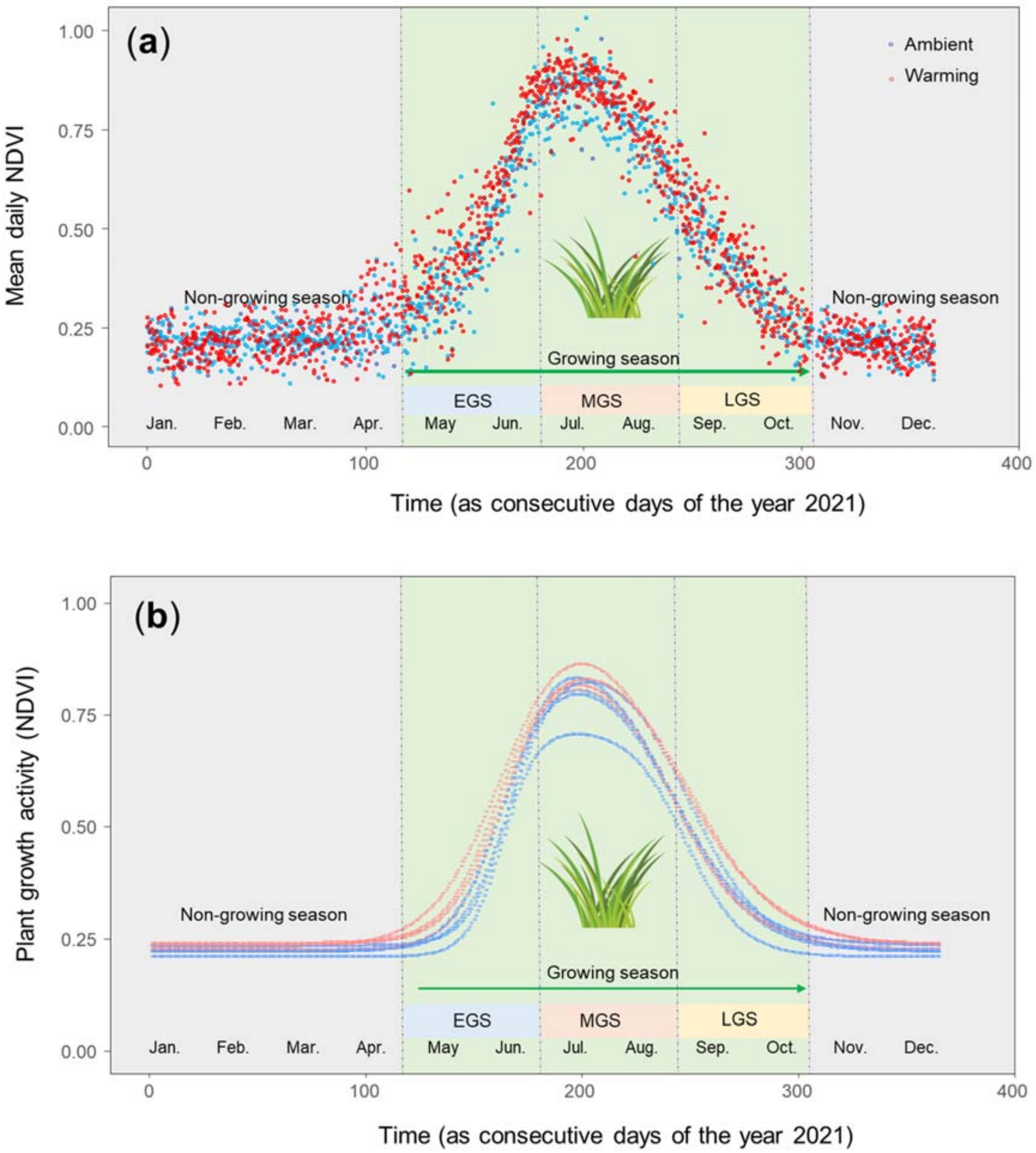

Supplementary Fig. 3 Plant growth is characterized by PhenoCam-derived NDVI data. Mean daily NDVI time series of phenological camera **(a)**, and NDVI time series with fitting a double logistic function **(b)** under ambient (with blue dots) and warming (with red dots) climates. Abbreviations: EGS = early growing season (spring = May–June), MGS = middle growing season (summer = July–August), and LGS = late growing season (autumn = September–October).

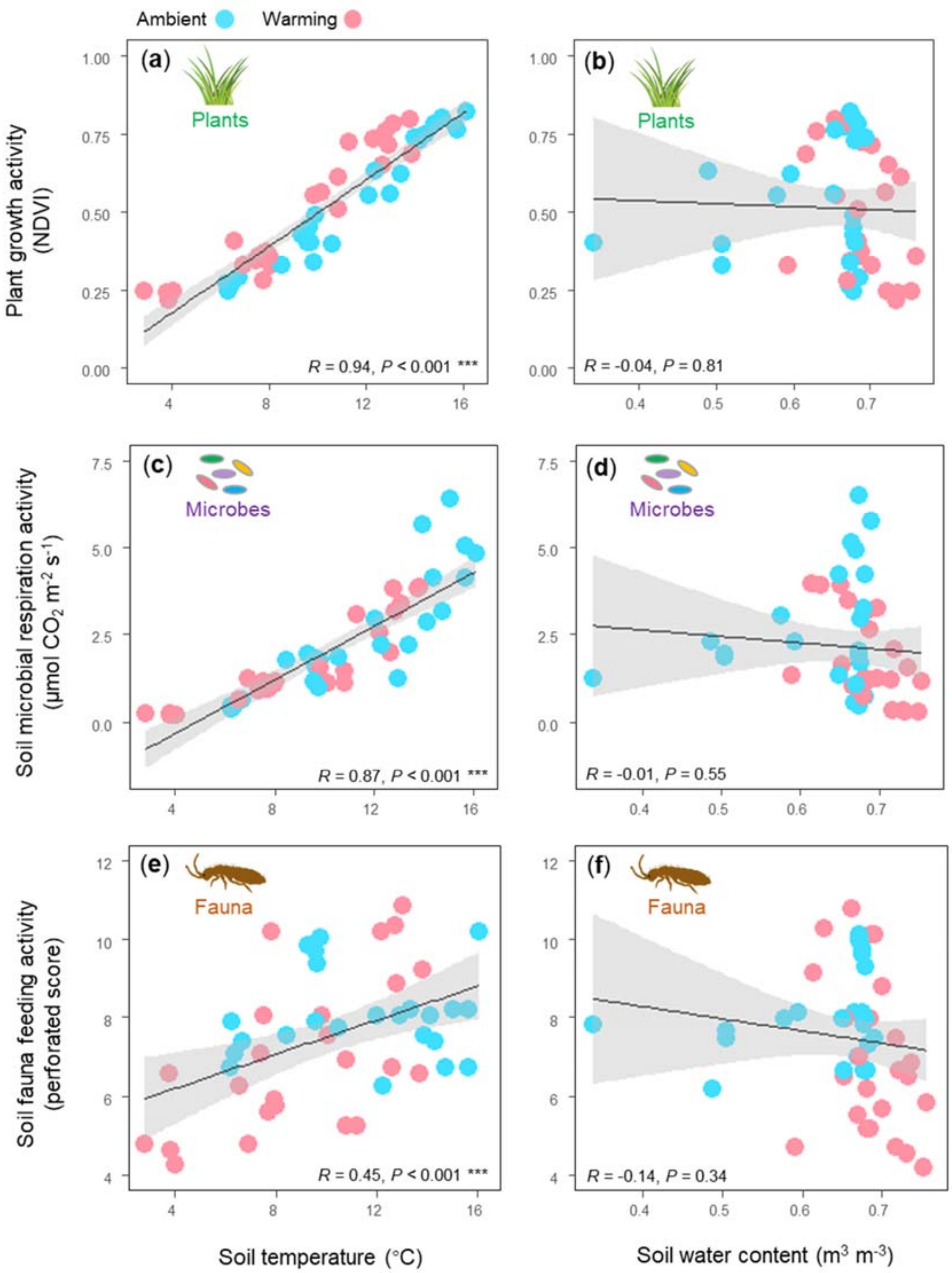

Supplementary Fig. 4 Relationship between alpine biological activities and soil abiotic conditions. Correlations (*R*) between soil abiotic factors (i.e., monthly mean temperature and water content at 5 cm soil depth) and plant growth activity **(a-b)**, and soil microbial respiration activity **(c-d)**, and soil fauna feeding activity **(e-f)**. The statistical tests are two-sided, and significant effects are indicated by *** = $P < 0.001$. Lines and error bands depict the best-fit trendline and the 95% confidence interval of the linear regression, respectively. Points represent biologically independent samples (n = 48; blue and red points represent ambient and warming climates, respectively).

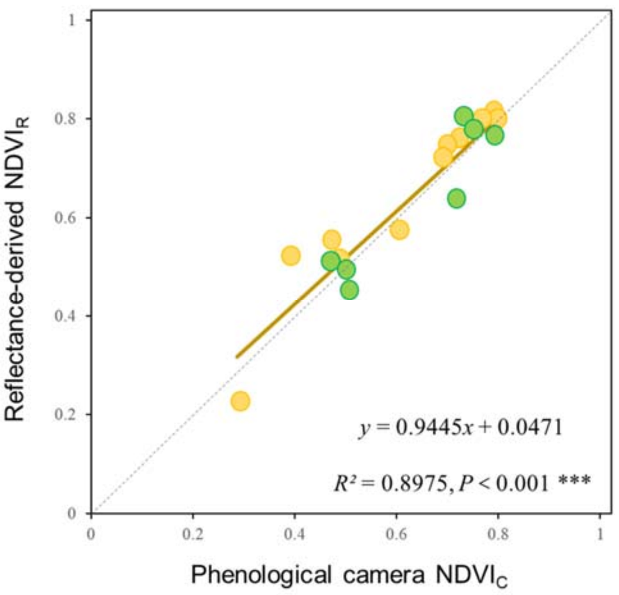

Supplementary Fig. 5 Linear regression of spectral reflectance-derived $NDVI_R$ and digital number-derived $NDVI_C$. The phenological camera was installed in 2018 and has been in operation for five years. The statistical test are two-sided, and significant effects are shown indicated by *** = $P < 0.001$. Lines depict the best-fit trendline of the linear regression, and points represent independent sampling plots (n = 18). Specifically, a total of 18 plots was measured during the growing seasons of 2018 (n = 11, in yellow) and 2019 (n = 7, in green).

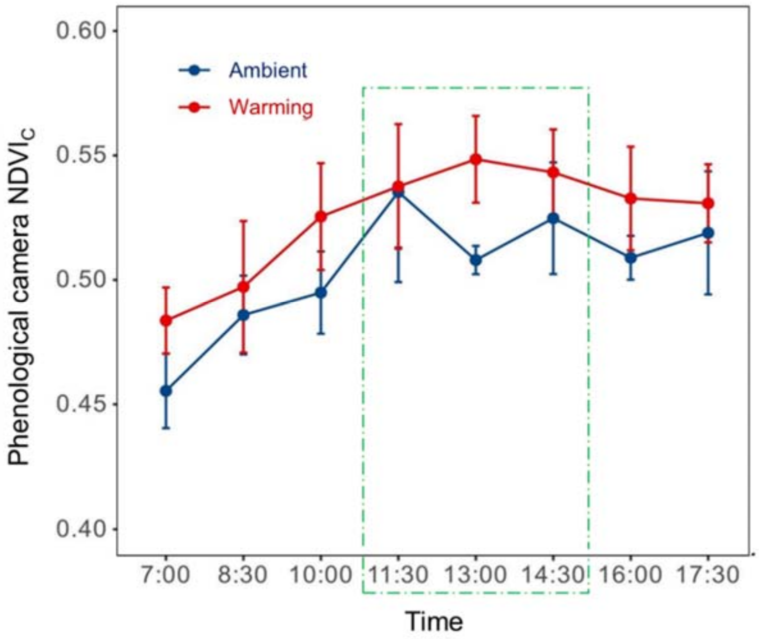

Supplementary Fig. 6 NDVI sub-daily series. The PhenoCam shot and retrieved eight NDVI values (at 7:00, 8:30, 10:00, 11:30, 13:00, 14:30, 16:00, 17:30) within a day for each plot. Based on the NDVI sub-daily series, daily maximum NDVI values (from 11:30 to 14:30) were used to calculate a mean daily maximum NDVI. The data shown are mean values ± SD.

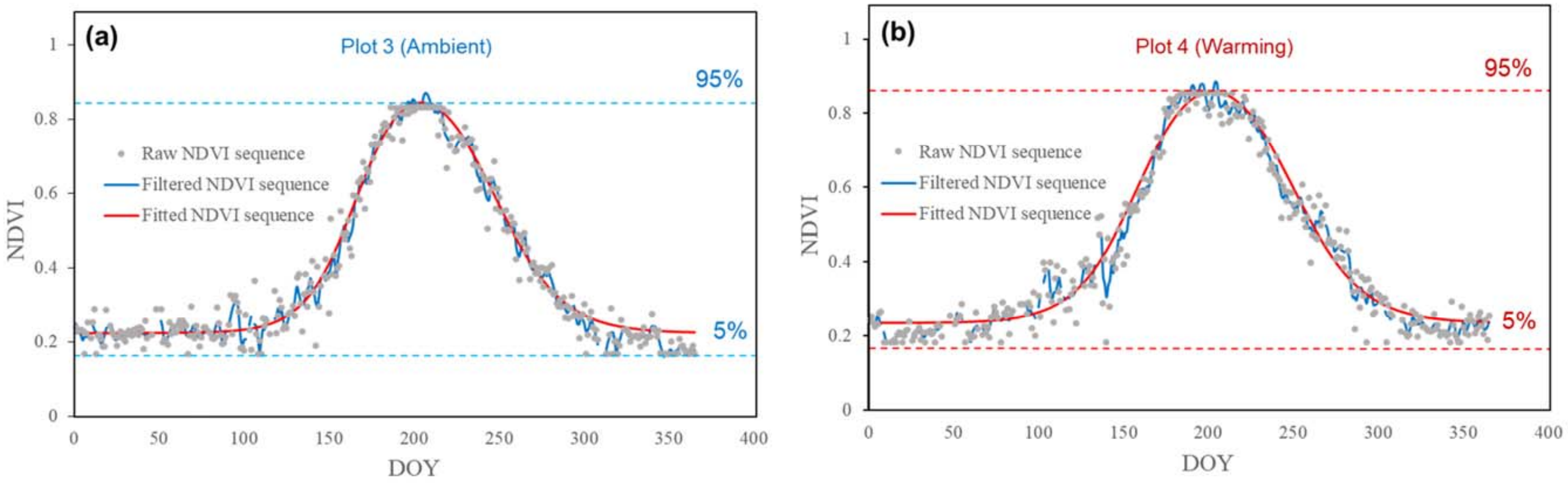

Supplementary Fig. 7 Comparison for the raw, filtered, and fitted NDVI sequences. These NDVI sequences are shown in both **(a)** ambient (plot 3) and **(b)** warming (plot 4) climates.

Supplementary Table 1 A brief flowchart of NDVI data processing. Note: taking plot 3 and (paired) plot 4 (within block 2) to represent the ambient and the warming plots, respectively.

Description	Reference figures
**<u>Step 1</u>**: Gaining phenological camera $NDVI_C$ raw data.	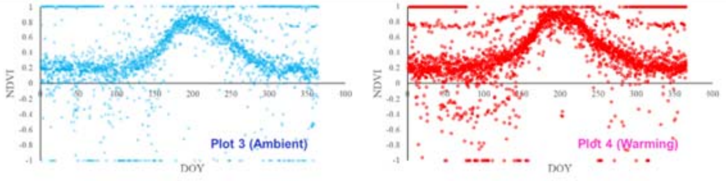
**<u>Step 2</u>**: Using a linear regression between Spectral reflectance $NDVI_R$ and $NDVI_C$ (Supplementary Fig. 5) to correct $NDVI_C$ and gain normal NDVI values[51].	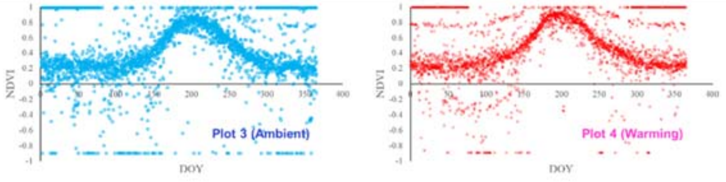
**<u>Step 3</u>**: Deleting the data points with NDVI ≤ 0 (usually due to snow cover in winter) and light deficiency-derived obvious outliers (like in cloudy days and early morning)[52].	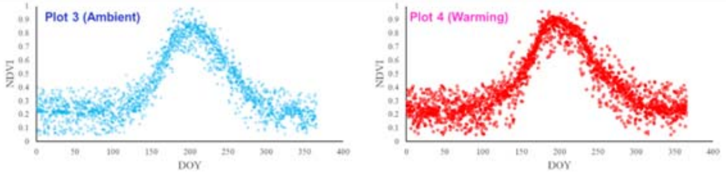
**<u>Step 4</u>**: Extracting the daily maximum NDVI values (11:30 - 14:30) (Supplementary Fig. 6) from the remaining values.	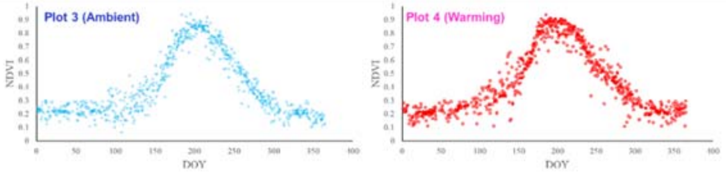
**<u>Step 5</u>**: Using the daily maximum NDVI values (11:30 - 14:30) to calculate mean daily NDVI values.	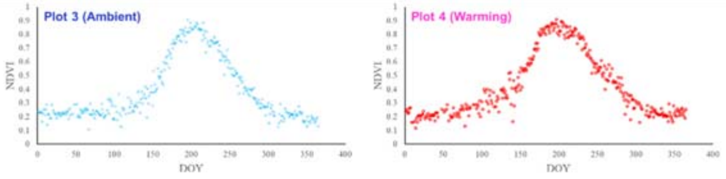
**<u>Step 6</u>**: Using the 95% and 5% quantiles as the upper and lower limits of NDVI sequences to constrain the mean daily NDVI values[51].	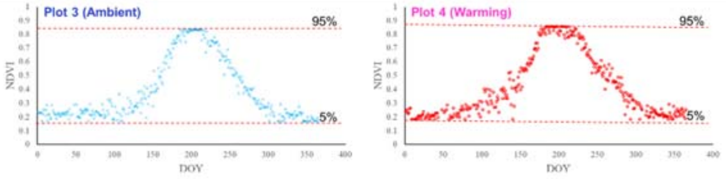
**<u>Step 7</u>**: Using the Savitzky-Golay filter in the R 'phenofit' package to filter and denoise NDVI sequences[53].	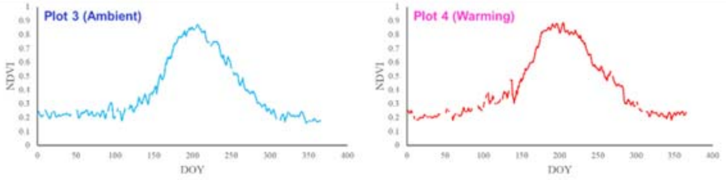
**<u>Step 8</u>**: Using the double logistic function to obtain the smooth seasonal dynamic curves of NDVI[54].	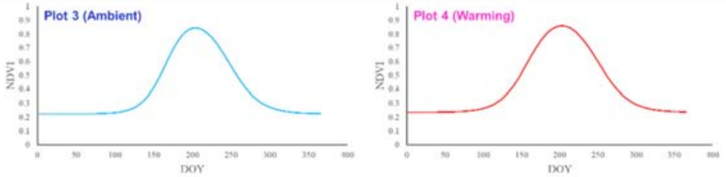
