# Supplementary material for: Experimental warming causes mismatches in alpine plant-microbe-fauna phenology
Source: Nat Commun. 2023 Apr 15;14:2159. doi: 10.1038/s41467-023-37938-3 (PMC10105701; doi:10.1038/s41467-023-37938-3)
Supplement: Supplementary file 3 — Reporting Summary [file 41467_2023_37938_MOESM3_ESM.pdf]

nature portfolio

Corresponding author(s): Biao Zhu
Last updated by author(s): Apr 4, 2023

Reporting Summary

Nature Portfolio wishes to improve the reproducibility of the work that we publish. This form provides structure for consistency and transparency in reporting. For further information on Nature Portfolio policies, see our Editorial Policies and the Editorial Policy Checklist.

Statistics

For all statistical analyses, confirm that the following items are present in the figure legend, table legend, main text, or Methods section.

n/a	Confirmed	
☐	☒	The exact sample size (*n*) for each experimental group/condition, given as a discrete number and unit of measurement
☐	☒	A statement on whether measurements were taken from distinct samples or whether the same sample was measured repeatedly
☐	☒	The statistical test(s) used AND whether they are one- or two-sided<br>*Only common tests should be described solely by name; describe more complex techniques in the Methods section.*
☒	☐	A description of all covariates tested
☐	☒	A description of any assumptions or corrections, such as tests of normality and adjustment for multiple comparisons
☐	☒	A full description of the statistical parameters including central tendency (e.g. means) or other basic estimates (e.g. regression coefficient) AND variation (e.g. standard deviation) or associated estimates of uncertainty (e.g. confidence intervals)
☐	☒	For null hypothesis testing, the test statistic (e.g. *F*, *t*, *r*) with confidence intervals, effect sizes, degrees of freedom and *P* value noted<br>*Give P values as exact values whenever suitable.*
☒	☐	For Bayesian analysis, information on the choice of priors and Markov chain Monte Carlo settings
☒	☐	For hierarchical and complex designs, identification of the appropriate level for tests and full reporting of outcomes
☐	☒	Estimates of effect sizes (e.g. Cohen's *d*, Pearson's *r*), indicating how they were calculated

Our web collection on statistics for biologists contains articles on many of the points above.

Software and code

Policy information about availability of computer code

Data collection	No software was used to collect data in this study.
Data analysis	Statistical analyses were performed using the statistical program R (version 4.2.2), and the R package 'lme4', 'multcomp', and 'phenofit'. Details were reported in the Statistical analysis section of the Methods.

For manuscripts utilizing custom algorithms or software that are central to the research but not yet described in published literature, software must be made available to editors and reviewers. We strongly encourage code deposition in a community repository (e.g. GitHub). See the Nature Portfolio guidelines for submitting code & software for further information.

Data

Policy information about availability of data

All manuscripts must include a data availability statement. This statement should provide the following information, where applicable:

- Accession codes, unique identifiers, or web links for publicly available datasets
- A description of any restrictions on data availability
- For clinical datasets or third party data, please ensure that the statement adheres to our policy

The data that support the findings of this study are available under CC-BY 4.0 license from Figshare. Data on the biological activities (i.e., plant growth, soil microbial respiration, soil fauna feeding), and soil abiotic conditions (i.e., soil temperature, soil moisture) are available from Figshare: https://doi.org/10.6084/

m9.figshare.22357921.v2; data on the other explanatory variables (i.e., plant biomass, soil microbial biomass carbon, soil fauna biomass) are available from Figshare: https://doi.org/10.6084/m9.figshare.22358158.v1; Data on the initial soil chemical properties (i.e., soil organic carbon, extractable organic carbon, total nitrogen, extractable total nitrogen, inorganic nitrogen, ammonium nitrogen, nitrate nitrogen, pH value) are available from Figshare: https://doi.org/10.6084/m9.figshare.22358254.v1.

Human research participants

Policy information about studies involving human research participants and Sex and Gender in Research.

Reporting on sex and gender	The study did not involve human research participants.
Population characteristics	The study did not involve human research participants.
Recruitment	The study did not involve human research participants.
Ethics oversight	The study did not involve human research participants.

Note that full information on the approval of the study protocol must also be provided in the manuscript.

Field-specific reporting

Please select the one below that is the best fit for your research. If you are not sure, read the appropriate sections before making your selection.

☐ Life sciences ☐ Behavioural & social sciences ☒ Ecological, evolutionary & environmental sciences

For a reference copy of the document with all sections, see nature.com/documents/nr-reporting-summary-flat.pdf

Ecological, evolutionary & environmental sciences study design

All studies must disclose on these points even when the disclosure is negative.

Study description	We conduced our study at the Haibei Alpine Grassland Ecosystem Research Station, utilizing a whole-soil-profile warming experiment in an alpine meadow on the Qinghai-Tibetan Plateau, China (37°29'-37°45' N, 101°12'-101°23' E, 3200 m a.s.l.). The experiment consists of 4 blocks. Each block contains two circular plots (with a diameter of 3.5 m), one of which is subjected to warming climate, while the other one is under ambient climate (Control). That is, each two plots (1 warmed plot + 1 ambient plot) are nested into one block, there are therefore 4 replicates for each climate treatment.
Research sample	For each plot, we collected plant NDVI data, and data on heterotrophic respiration and fauna activity every month (from May to October, 2021) to investigate the effects of warming on the activities of plant growth, microbial respiration, and fauna feeding. Additionally, in mid-August 2021, we measured plant biomass (shoot and root, g m-2), soil microbial biomass (mg kg-1), and soil fauna biomass (mg m-2) as potential explanatory variables to support our main data.
Sampling strategy	Sampling strategy was determined together with the experimental design. Four biological independent replicates (n = 4) for each climate treatment were sampled monthly (from May to October, 2021). These 24 samples/data for each biological (plant/soil microbe/soil fauna) activity are sufficient for measuring variation and evaluating differences in and between ambient and warming climates using statistic tests, such as linear mixed effects models.
Data collection	We collected the data of (i) soil temperature and soil water content by a custom-made thermistor and a PR2/6 sensor (Delta-T Devices Ltd., UK) at 10-minute intervals, respectively; (ii) plant NDVI data by a PhenocCam; (iii) soil microbial respiration by a PVC collar method; (iv) soil fauna feeding activity by bait-lamina method.
Timing and spatial scale	We conducted the date and samples during the growing season of 2021 (from May to October), i.e., three years after the beginning of the experiment. The aim of this study was to investigate the plant growth, soil microbial respiration, and fauna feeding across months of the alpine growing season. To do so, we collected monthly for each responding variable in each plot Specifically, we collected the data of PhenoCam-derived Normalized Difference Vegetation Index NDVI, soil microbial respiration (to a soil depth of 60 cm), soil fauna feeding (to a soil depth of 10 cm) every month from May to October 2021. Additionally, we measured mean monthly soil temperature and water content at 5 cm soil depth from May to October 2021.
Data exclusions	No data were excluded from the analyses.
Reproducibility	Each climate treatment was successfully replicated 4 times. This replicate number was accurately for identifying differences between ambient climate and warming climate.
Randomization	Within block, plots were randomly assigned to each of two temperature treatments: ambient or warmed.
Blinding	N/A

Did the study involve field work? [x] Yes [ ] No

Field work, collection and transport

Field conditions	The climate of this study area is continental monsoon. The average annual air temperature is about 1.1°C, and the highest temperature occurs in July or August, and the lowest temperature occurs in January. Average annual precipitation over the last three decades was about 485 mm, with most annual precipitation (84%) falling from May to September.
Location	The Qinghai-Tibetan Plateau, China (37°29'-37°45' N, 101°12'-101°23' E).
Access & import/export	Soil samples were collected with explicit permission from national and local authorities.
Disturbance	After taking the soil samples, the holes were back-filled to minimize the disturbance to the plots.

Reporting for specific materials, systems and methods

We require information from authors about some types of materials, experimental systems and methods used in many studies. Here, indicate whether each material, system or method listed is relevant to your study. If you are not sure if a list item applies to your research, read the appropriate section before selecting a response.

Materials & experimental systems

n/a	Involved in the study
[x]	[ ] Antibodies
[x]	[ ] Eukaryotic cell lines
[x]	[ ] Palaeontology and archaeology
[ ]	[x] Animals and other organisms
[x]	[ ] Clinical data
[x]	[ ] Dual use research of concern

Methods

n/a	Involved in the study
[x]	[ ] ChIP-seq
[x]	[ ] Flow cytometry
[x]	[ ] MRI-based neuroimaging

Animals and other research organisms

Policy information about studies involving animals; ARRIVE guidelines recommended for reporting animal research, and Sex and Gender in Research

Laboratory animals	The study did not involve laboratory animals.
Wild animals	The study involved soil invertebrates, but did not involve vertebrate animals.
Reporting on sex	N/A
Field-collected samples	Soil invertebrates were collected in experimental plots to investigate their total biomass.
Ethics oversight	No ethical approval or guidance was required to investigate soil invertebrates.

Note that full information on the approval of the study protocol must also be provided in the manuscript.
